# Numerical Analysis of the Effect of Retaining Ring Structure on the Chemical Mechanical Polishing Abrasive Motion State

**DOI:** 10.3390/ma16010062

**Published:** 2022-12-21

**Authors:** Siqi Zhang, Yiran Liu, Weimin Li, Jun Cao, Jiaye Huang, Lei Zhu, Zijun Guan

**Affiliations:** 1 School of Microelectronics, Shanghai University, Shanghai 200444, China; 2 Shanghai Institute of IC Materials, Shanghai 200050, China; 3 Shanghai Institute of Microsystem and Information Technology, Chinese Academy of Sciences, Shanghai 200050, China; 4 School of Mechanical and Power Engineering, East China University of Science and Technology, Shanghai 200237, China

**Keywords:** chemical mechanical polishing, abrasive, retaining ring, size of grooves, quantity of grooves, numerical simulation

## Abstract

Optimizing the retaining ring structure can improve the quality of Chemical Mechanical Polishing (CMP). This study establishes a two-dimensional Computational Fluid Dynamics-Discrete Element Method (CFD-DEM) model, while the model is validated by experiments. The results graphically demonstrate the influence of the retaining ring groove design on the motion of the slurry abrasive particles. The size of the retaining ring groove appears to have a threshold value, above which the abrasives start to have significant distribution in the wafer region. As the groove size continues to increase, the number of abrasives entering the ring increases abruptly and oscillates at specific nodes. The abrasive transfer rate increases with the number of grooves in the early stage but reaches a limit at a certain number of grooves. Meanwhile, the retaining ring position affects the transfer of the abrasives. This study provides a base for optimizing the design of retaining rings.

## 1. Introduction

CMP is the process of achieving global flattening in advanced semiconductor manufacturing [1,2,3,4,5,6,7,8,9,10,11,12,13]. The ever-decreasing chip feature size poses more stringent requirements on the flatness of the single-crystal silicon wafers [14,15]. It is important to study and understand in more detail every factor that may impact the local polishing rate.

In the CMP process, the wafer is attached to the polishing head and confined by the retaining ring. Then, it is pressed face-down onto the polishing pad. The head carrying the wafer and the pad carrying the slurry rotate around their respective centers. The slurry, which is composed of liquid and abrasive particles, is carried by the rotating pad to the polishing gap between the wafer and the pad [16,17]. The wafer surface reacts chemically with slurry to generate a surface layer that is relatively easy to remove, which is then mechanically grounded off by the abrasives [18,19].

The retaining ring groove design had significant impacts on improving material removal rate (MRR), flatness, and providing a way to exchange the slurry with the external polishing slurry into the pad-wafer interface [20,21,22]. For a high-volume manufacturing commercial CMP device, the movement of abrasives flowing into the wafer region cannot be directly measured as the wafer and retaining ring assembly are non-transparent. Recently, numerical simulations have paid more and more attention to device and process optimization. Since the CMP process is not completely understood, numerical models based on mechanisms are helpful for the understanding of the motion behavior of slurry and abrasives [23].

**Figure 1:**
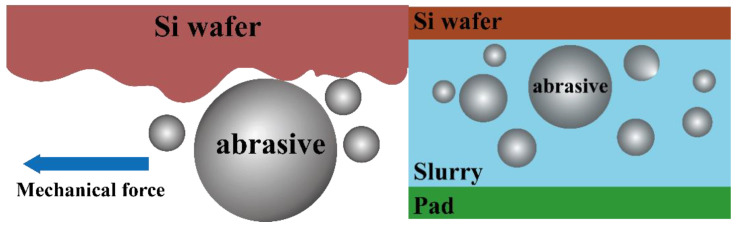
Schematic diagram of the mechanical effect of abrasives.

Some studies have shown that the flat surface of the wafer can be obtained by repeatedly sliding, rolling, and squeezing the abrasives in slurry [15,24,25,26], as shown in Figure 1. Therefore, the movement state of abrasives in the slurry flow is one of the most fundamental factors affecting the wear of the wafer substrate. Furthermore, the groove design of the retaining ring provides a way to exchange the slurry at the bottom of the wafer with the external polishing slurry. Several experiments and numerical simulations have been reported to investigate the slurry distribution in the polishing gap and the effect of the retaining ring on the slurry distribution in the polishing gap. Philipossian et al. applied the Dual Emission Ultraviolet Enhanced Fluorescence (DEUEF) technique, which projects UV light to the photoreceptor region under the wafer to excite the dye and uses two Photometrics Sensys Cameras to capture the fluorescence intensity emitted by the dye. The fluorescence intensity is converted into the slurry film thickness between the wafer and the polishing pad after calibration [20,22,27,28]. This technique was used to study the effect of the retaining ring groove design on the slurry film thickness between polishing gaps [20,22,28,29]. Bengochea et al. studied the effect of retaining ring grooves on polishing gap film thickness and slurry bow wave [20,30]. Moinpour et al. also studied slurry distribution in the polishing gap using different excitation fluorescent dyes [31,32]. These studies on polishing gaps have equipment limitations. Moreover, the abrasives in the slurry are nanoscale, and the method of capturing the fluorescence intensity of the dye cannot characterize the motion of the abrasives. Other methods are also explored to study the motion of the abrasives. Nguyen et al. investigated the effect of the polishing pad and wafer speed on the distribution of abrasives in slurry based on multiphase flow and discrete phases [33]. Ship-Peng Lo et al. established a two-dimensional axisymmetric static finite element model of wafers and polishing pads to investigate the relationship between the retaining ring and the wafer surface loading [34]. Previous studies focused on the local movement of the abrasives, but there is no published work studying the effect of retaining ring structure on the macro distribution of abrasive particles on the whole pad, which consequently affects the wafer scale polishing performance.

In this paper, a two-dimensional multi-physics model is established using the Finite Element Method and the Discrete Element Method, aiming to study the effect of the retaining ring design on the abrasive movement during the CMP process. The effects of the retaining ring groove size on the abrasive transfer rate are investigated, and the periodic variation patterns of abrasive transfer through the grooves are observed. On this basis, the number of grooves can be changed to optimize the distribution of abrasives in the wafer region and to improve the flatness of the wafer.

## 2. Materials and Methods

### 2.1. Basic Theory and Model Geometry

**Figure 2:**
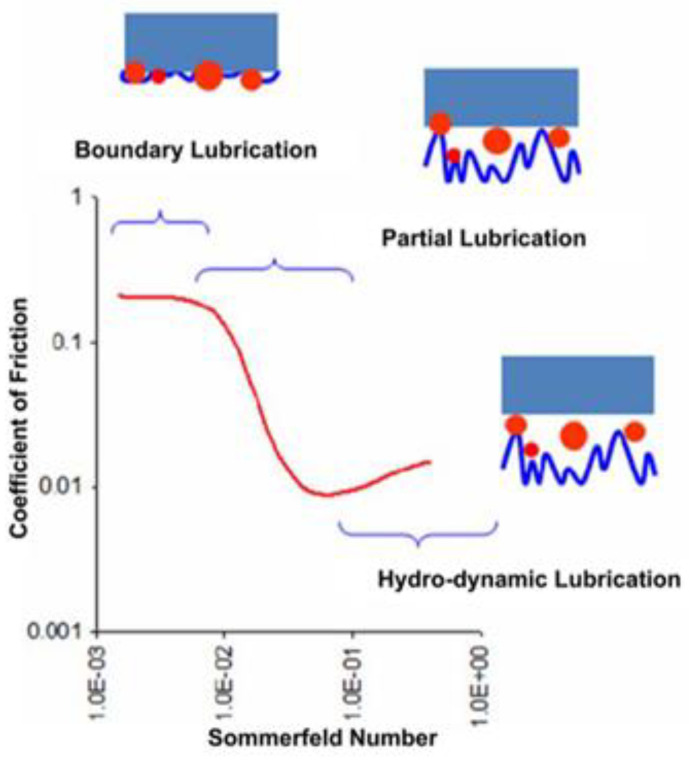
Stribeck curve of the CMP process [38].

In the CMP procedures, there are three contact models that exist depending upon the material and process parameters, namely (i) boundary lubrication, (ii) partial lubrication, and (iii) hydrodynamic lubrication, as depicted in Figure 2. The lubrication mode between the pad and the wafer can be defined based on the Sommerfeld number of the Stribeck curve. The Sommerfeld number is defined as [35]:

(1) $$So=\frac{\mu V}{p\delta }$$

where μ is the slurry viscosity, V is the relative moving speed, p is the applied pressure, and δ is the effective fluid thickness between pad and wafer [36]. With a high rotation speed and a small load, which is in line with the model parameters in this work, the wafer will glide on a fluid film without directly touching the pad. A thick slurry film (20–50 μm) separating the wafer from the pad reduces direct contact between the pad and wafer, thereby reducing scratching of the wafer surface [37]. The distribution of the abrasives in contact with the wafer is influenced by the flow field of the liquid layer near the wafer surface when material removal occurs. The existence of the layer is an initial assumption for the analysis, and evaluating the validity of the assumption is the primary focus.

**Figure 3:**
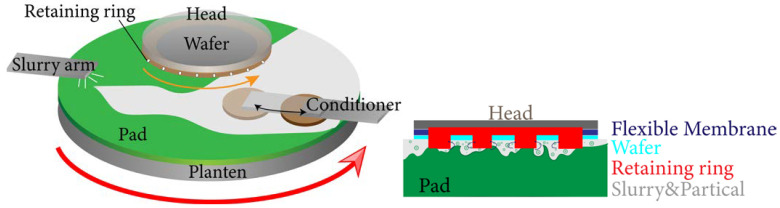
Schematic diagram of CMP process.

In a CMP process, there are two main sources of polishing slurry in the polishing gap: one part already exists on the polishing pad before the polishing head is pressed down, and the other part flows through the grooves of the retaining ring. To simplify the modeling, it is assumed that the abrasives in the polishing gap before the polishing head is pressed down are not affected by the retaining ring and are in a stable state. Therefore, this part of the abrasives can be ignored in the modeling of the abrasive distribution, and the focus of this study was set on the abrasives passing through the grooves of the retaining ring. Before the abrasives entered the polishing gap, they were scraped flat by the conditioner, as shown in Figure 3. Therefore, the abrasives are assumed to be evenly distributed and pressed into the near-surface pores of the pad, thus do not move laterally relative to the pad. For simplicity, the word “slurry” in the rest of this paper means the part of the slurry that can move freely on the pad surface.

**Figure 4:**
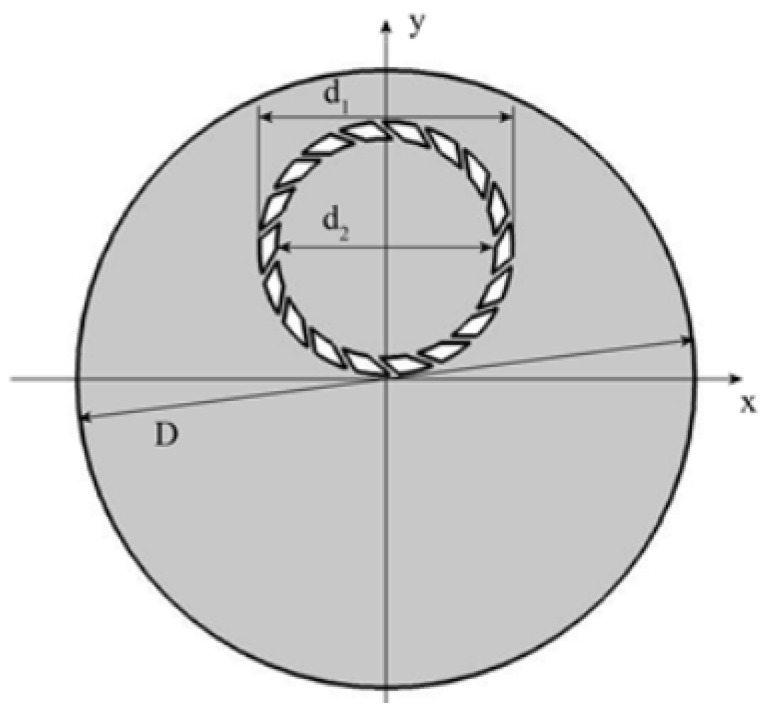
CMP model geometric structure diagram.

Based on the general polishing process and the parameters of the polishing pad, retaining ring, and slurry, a two-dimensional finite element model is constructed, as shown in Figure 4. In this model, D is the diameter of the polishing pad, d_1_ is the outer diameter of the retaining ring, and d_2_ is the inner diameter. The surface slurry is distributed throughout the pad and flows freely with the rotation of the pad, the wafer, and the retaining ring. The nano-sized spherical abrasives are uniformly dispersed in the slurry except when colliding with an object. Collisions between abrasives are not considered in this work.

### 2.2. Governing Equation

COMSOL Multiphysics 6.0 is used to conduct Finite Element Analysis. The slurry flow in the chemical mechanical polishing device is relatively turbulent, so this paper uses the k-epsilon turbulent model and the particle tracing model in a turbulent flow. The model is based on the following assumptions:

1. Slurry and abrasives are incompressible;
2. The gap between the wafer and the polishing pad is the near-surface layer of the wafer. The velocity of the liquid layer on the wafer surface is approximately that of the wafer [33];
3. The total amount of slurry and abrasives is in a stable state.

The continuity equation and momentum equation describing the flow process of the polishing slurry are as follows:

Continuity equation:

(2) $$\frac{\partial \rho }{\partial t}+\rho 𝛻\cdot u=0$$

Momentum equation:

(3) $$\frac{\partial \rho }{\partial t}+\rho \left(u\cdot 𝛻\right)u=𝛻\cdot \left(-pI+K\right)+F$$

(4) $$K=\left(\mu +\mu_{T}\right)\left(𝛻u+{\left(𝛻u\right)}^{T}\right)$$

where *u* is the slurry velocity, *p* is the slurry pressure, *ρ* is the slurry density, *µ* is the slurry dynamic viscosity, *µ_T_* is the eddy viscosity, and *F* is the additional force acting on the slurry.

Among them, *F* represents the equivalent replacement force of polishing pad rotation, which is composed of Coriolis force and centrifugal force:

(5) $$F=-\rho \left(2\vec{\omega }\cdot u+\vec{\omega }\cdot \vec{\omega }\cdot \vec{r}\right)\cdot \hat{i}$$

where *ρ* is the slurry density, *u* is the slurry velocity, *ω* is the angular velocity of circular motion, and *r* is the distance between the abrasive and the center of the circle.

The k-epsilon equation is as follows:

(6) $$\frac{\partial \rho k}{\partial t}+\rho \left(u\cdot 𝛻\right)k=𝛻\left[\left(\mu +\frac{\mu_{T}}{\sigma_{k}}\right)𝛻k\right]+P_{k}-\rho \varepsilon$$

(7) $$\frac{\partial \rho \varepsilon }{\partial t}+\rho \left(u\cdot 𝛻\right)\varepsilon =𝛻\cdot \left[\left(\mu +\frac{\mu_{T}}{\sigma_{\varepsilon }}\right)𝛻\varepsilon \right]+C_{\varepsilon 1}\frac{\varepsilon }{k}P_{k}-C_{\varepsilon 2}\rho \frac{\varepsilon^{2}}{k}$$

(8) $$\mu_{T}=\rho C_{\mu }\frac{k^{2}}{\varepsilon }$$

(9) $$P_{k}=\mu_{T}[𝛻u:\left(𝛻u+\left(𝛻u{)}^{T}\right)\right]$$

where *ε* is the turbulent dissipation rate, *k* is the turbulent kinetic energy, *σ_k_* and *σ_ε_* are the respective turbulent Prandtl numbers of *σ* and *k*, *µ_T_* the eddy viscosity coefficient, and *P_k_* is the generation term of turbulent kinetic energy caused by the average velocity gradient.

In order to consider the rotation of both the pad and the polishing head, the model is calculated in two reference frames. One is a moving zone consisting of the retaining ring and the internal flow region, and the other is a stationary zone for the region outside the retaining ring.

The computational domain with respect to the moving frame is defined such that an arbitrary point in the CFD domain is located by a position vector from the origin of the moving frame. The fluid velocity can be transformed from the stationary frame to the moving frame based on the following equation:

(10) $$\vec{\nu_{r}}=\vec{\nu }-\left(\vec{\nu_{t}}+\vec{\omega }\vec{r}\right)$$

where $\vec{\nu_{r}}$ is the relative velocity (the velocity viewed from the moving zone), $\vec{\nu }$ is the absolute velocity (the velocity viewed from the stationary zone), $\vec{\nu_{t}}$ is the translational velocity, and $\vec{\omega }$ is the angular velocity. It is worth noting that both $\vec{\omega }$ and $\vec{\nu_{r}}$ can be functions of time.

Based on the turbulence model and the multiple moving frames, the flow rate of the slurry can be obtained, and the movement of the abrasives can be analyzed. The abrasives are completely immersed in the slurry. During the rotation process, the turbulent flow produces a drag force on the abrasives, which drives the movement of the abrasives.

The movement of the abrasives in the flow field can be expressed as:

(11) $$\frac{d}{dt}\left(m_{p}V\right)=F_{D}$$

where *v* and *m_p_* represent the speed and the mass of the abrasive, respectively, and *F_D_* is the drag force on the abrasives.

The drag force on the abrasives is related to the size, mass, and velocity of the abrasives, as well as the velocity and dynamic viscosity of the slurry, and can be calculated by the following formula:

(12) $$F_{D}=\frac{1}{\tau_{p}}m_{p}\left(u-v\right)$$

where *τ_p_* is the abrasive speed response time indicating how fast the abrasive is accelerated to the slurry velocity, which is expressed by the formula:

(13) $$\tau_{p}=\frac{\rho_{p}d_{p}^{2}}{18\mu }$$

where *ρ_p_* is the abrasive density, *d_p_* is the abrasive diameter, and *µ* is the hydrodynamic viscosity.

The flow in the slurry is turbulent, so the reaction of the abrasive colliding with the wall surface of the retaining ring is a diffuse reflection.

### 2.3. Boundary Conditions

For the coupled multi-physical field model of slurry-abrasive interaction established in this paper, the boundary conditions can be expressed as:

1. Based on the high rotational speed, the contact mode among the pad, slurry, and wafer can be defined as hydrodynamic lubrication, in which case the pad and the wafer are not in direct contact but separated by the slurry layer. Therefore, the slurry flows freely in the model;
2. Initial value (*u* is the fluid velocity, *v* is the abrasive velocity):
  *u* = 0; *v* = 0 (14)
3. Wall condition: In this paper, the wall is defined as a non-slip boundary, and the flow velocity at the wall surface is 0 m·s^−1^. During the flowing process, velocity gradually increases to the main flow velocity with the increase of the distance from the wall surface;
4. Rotation domain setting.

In this paper, the rotation of the retaining ring is realized by setting the rotating domain. The area of the rotating domain is a circle with a center of (0,180 mm) and a diameter of 356 mm.

### 2.4. Grid Independence Verification

**Figure 5:**
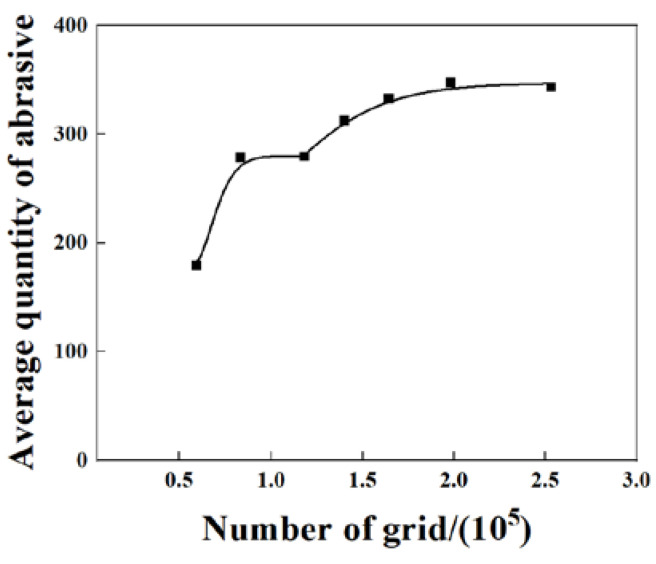
The effect of grid number on the number of abrasives.

The grid density has an impact on the computational results, and the grid density reaches a threshold value to keep the influence of the grid at a low level. Therefore, a mesh-independent verification of the model is required to determine the non-correlation between the number of meshes used in the model and the results obtained from the calculations. As the abrasive transmission through the retaining ring is of the main concern in this paper, the average number of the abrasives in the first cycle is selected as the basis for judgment, as shown in Figure 5. The number of grids is increased, with 60,000 grids as the starting point. When the number of grids is below 100,000, the average abrasive quantity is strongly influenced by the grids. As the number of grids reaches about 150,000, the average abrasive quantity tends to be smooth. Therefore, the 160,000 grids model in this paper can achieve high accuracy while saving computational costs.

### 2.5. Calculation Parameter

In the CMP retaining ring model, Table 1 presents the parameters used for the calculation.

The retaining ring groove design in the simulation is shown in Table 2.

## 3. Experimental

The polishing data was obtained using a 300 mm wafer polishing machine Universal—300Dual(HWATSING, Tianjin, China). SiO_2_ abrasive slurry (NP7050, NITTA DuPont, Osaka, Japan) was used for polishing a 300 mm silicon bare wafer to evaluate the effect of the retaining ring groove. A polyurethane polishing pad (SPM-3100, NITTA DuPont, Japan) was used in the experiment. The speed of the polishing head/pad was set to 120/120 rpm, and the flow rate of the slurry was 300 mL/min. The retaining ring structure used in the experiments is shown in Table 3.

**Figure 6:**
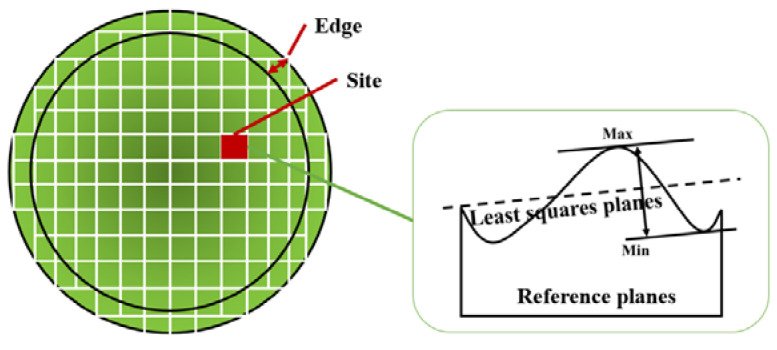
Definition of ESFQR.

The MRR and ESFQR (Edge Site Front Least Squares Range) were measured to verify the simulation model in a real manufacturing process. The MRR and ESFQR of wafers were measured by Bare Wafer Geometry Metrology Systems (WaferSight2+, KLA Tencor, Milpitas, CA, USA). MRR is defined as the amount of material removed per minute, while ESFQR is used to characterize the flatness of the wafer surface. As shown in Figure 6, the entire surface of the wafer is divided into multiple sites, and thickness variation is measured at each of these sites. ESFQR defines the maximum and minimum value difference in their respective site before the Edge (1 mm) [39], while Δ ESFQR refers to the improvement of the wafer surface ESFQR by CMP, which is defined as the difference between the ESFQR before and after polishing.

## 4. Results and Discussion

### 4.1. Effect of Retaining Ring Groove Width on Abrasive Transfer Rate

The abrasive transfer rate is defined as the ratio of the amount of abrasive moving to the polishing gap to the total global abrasive as follows:

(15) $$P_{t}=\frac{N_{a}}{N}$$

where *N_a_* is the number of abrasives entering the polishing gap, and *N* is the total number of abrasives in the polishing pad.

In order to study the effect of the groove width of the retaining ring on the ability of the abrasive to enter the polishing gap through the groove, the abrasive transfer rate was calculated with a different groove width of the retaining ring.

**Figure 7:**
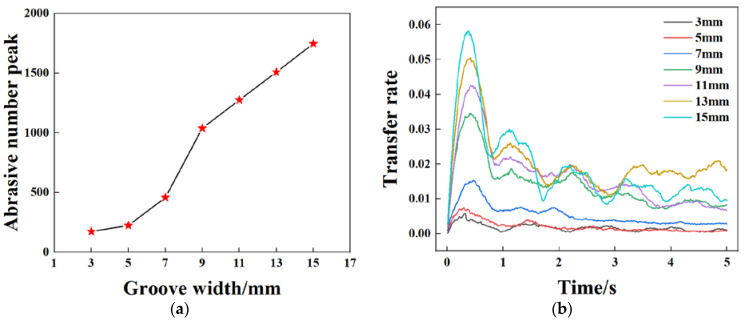
Abrasive transfer rate curves with different retaining ring groove widths. (**a**) Abrasive number peak as a function of groove width, (**b**) abrasive transfer rate under different groove width.

Figure 7 shows the transfer rate of abrasives from the outside of the retaining ring to the wafer area for different groove widths. The results show that under the constant parameter conditions, the abrasive transfer rate has a periodic bell-shaped curve, especially between 0–0.8 s. The transfer rate curve increases quickly and peaks at about 0.4 s at a groove size of 9 mm and higher. In the 0–0.8 s period, when the groove size increases from 3 mm to 5 mm, the number of abrasives in the rotation domain increases by 28%. When the groove size is as large as 7 mm, the transfer rate increases by 104%. When the groove size increases from 7 mm to 9 mm, the transfer rate increases by 127%. When bigger than 9 mm, the increasing rate of abrasive transfer gradually slows down. When the groove width increases from 9 mm to 11 mm, 13 mm, and 15 mm, the increment of transfer rate slows down to 23%, 18%, and 15%, respectively.

**Figure 8:**
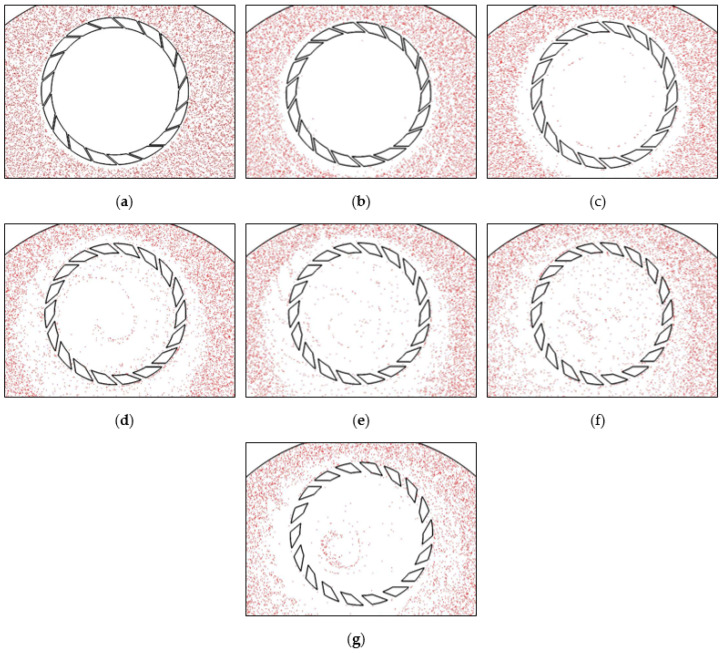
Abrasive distribution nephogram in polishing gap with different groove widths: (**a**) 3 mm, (**b**) 5 mm, (**c**) 7 mm, (**d**) 9 mm, (**e**) 11 mm, (**f**) 13 mm, (**g**) 15 mm.

In order to observe the distribution of the abrasives in the polishing gap, the abrasive movement is tracked and visualized at an interval of 0.01 s. Figure 8 shows the distribution of the abrasives in the wafer area after 5 s. The cloud atlas shows that the groove size not only affects the abrasive transport rate but also affects the abrasive distribution. When the retaining ring groove width is 3 mm and 5 mm, the abrasives do not enter the wafer area substantially. As the groove width reaches 7 mm, the abrasives entering through the grooves can reach the wafer area but hardly to the wafer center. At the groove widths of 9 mm, 11 mm, and 13 mm, the abrasive distribution is relatively uniform. The abrasive distribution is more uniform for the groove width of 13 mm than that for the widths of 9 mm and 11 mm. When the groove width is 15 mm, the abrasive distribution area is reduced, and the distribution is uneven.

**Figure 9:**
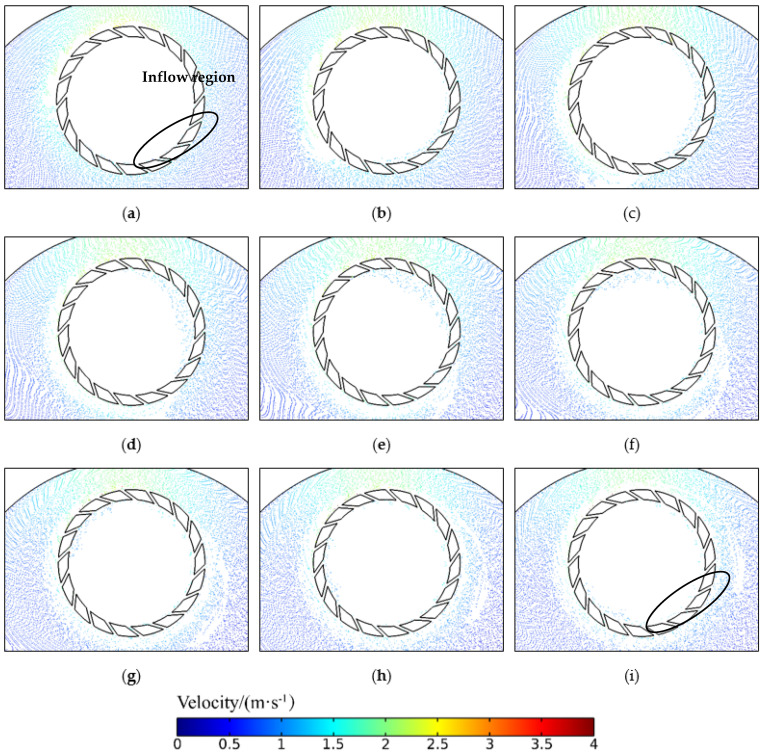
Abrasive entry and exit at different times: (**a**) 0.1 s, (**b**) 0.2 s, (**c**) 0.3 s, (**d**) 0.4 s, (**e**) 0.5 s, (**f**) 0.6 s, (**g**) 0.7 s, (**h**) 0.8 s, (**i**) 0.9 s.

It can be seen from the above that the groove size of the retaining ring has a large effect on the abrasive transfer rate. The abrasive transfer rate in Figure 7b shows a periodicity. This phenomenon shows that the abrasive flux entering the ring at certain positions and exiting at other positions are not in balance nor stable when the ring is pressed on the pad. Figure 9 shows the abrasive motion state during 0.1–0.9 s of the retaining ring with 18 7 mm grooves (Supplementary Materials). From 0 to 0.4 s, the abrasive transfer rate is in the rising phase. The abrasives enter the wafer region with a wave-like trend, indicating that the area at the lower part of the retaining ring to the upper right circular arc is the abrasive inflow region. The abrasive speed in the grooves in this region is approximately 1–1.5 m/s, which is less than the retaining ring speed of 120 rpm (approximately 1.9 m/s), under which circumstances the abrasives enter the grooves into the wafer region. During the period from 0.4 s to 0.8 s, the abrasive transport rate is in a decreasing phase. At this point, the abrasive wave in the wafer region is transferred to the area above the retaining ring to the left of the arc, implying that this area is the abrasives outflow area. In this area, the speed of the groove is 3–4 m/s, which is much faster than the speed of the retaining ring at 1.9 m/s. During this period, the abrasives enter the groove and escape from the wafer area. At the time of 0.9 s, the abrasive wave is transferred back to the bottom of the retaining ring, and the abrasive transfer rate curve steps into the second cycle. Due to the disturbance of the retaining ring rotation, the number of abrasives close to the abrasive inflow region cannot be replenished to the initial state. The abrasive transfer rate tends to decrease compared with the first cycle until it reaches a relatively stable state. From the above analysis, it can be deduced that the increase in groove size has a significant effect on the increase of the abrasive transfer rate in the first cycle, but at the same time, its effect on the abrasive in the outflow area should be considered.

### 4.2. Effect of Retaining Ring Groove Number on Abrasive Transfer Rate

Under the same process parameters, a retaining ring model with a groove width of 7 mm is selected as the reference since it is the size when the abrasives start to be distributed significantly. The number of grooves is varied to study the effect on the abrasive transfer rate.

**Figure 10:**
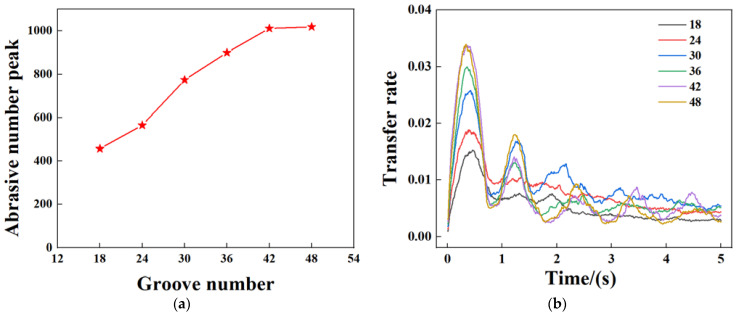
Variation curve of abrasive transfer rate under different groove numbers of 7 mm groove width retaining ring: (**a**) Abrasive number peak as a function of groove number, (**b**) abrasive transfer rate under different groove numbers.

Figure 10 shows the variation of the abrasive transfer rate with time for different numbers of grooves in the retaining ring. During the process from 0 s to the end of 5 s, the increase in the number of grooves enhances the communication inside and outside the wafer area, allowing more abrasive to pass through when the number of grooves is between 18–42, with the peak increase in transfer rate being 22.8%, 37.2%, 16.3%, and 12.3%, respectively. When the number of grooves is increased to 48, the transfer rate becomes stable. It can be observed that from the second cycle, the transfer rate peaks at the groove number of 30. It is assumed that further increasing the number of grooves contributes more to the abrasive exiting the retaining ring.

**Figure 11:**
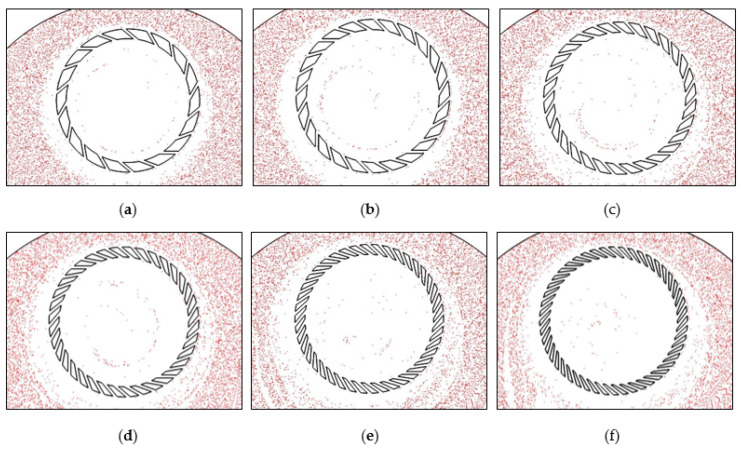
Abrasive distribution nephogram in polishing gap with different groove numbers: (**a**) 18, (**b**) 24, (**c**) 30, (**d**) 36, (**e**) 42, (**f**) 48.

In this section, the abrasives are tracked at a frequency of 0.01 s to observe the abrasive distribution in the polished area. Figure 11 shows the distribution of the abrasives at the end of the fifth second. The comparison shows that when 18, 24, 30, and 36 grooves designs are applied, the abrasive trajectory is spiral and relatively similar, and the overall distribution spreads toward the center of the wafer as the number of grooves increases. However, in the models with 42 and 48 grooves, the distribution of the abrasives becomes scattered.

**Figure 12:**
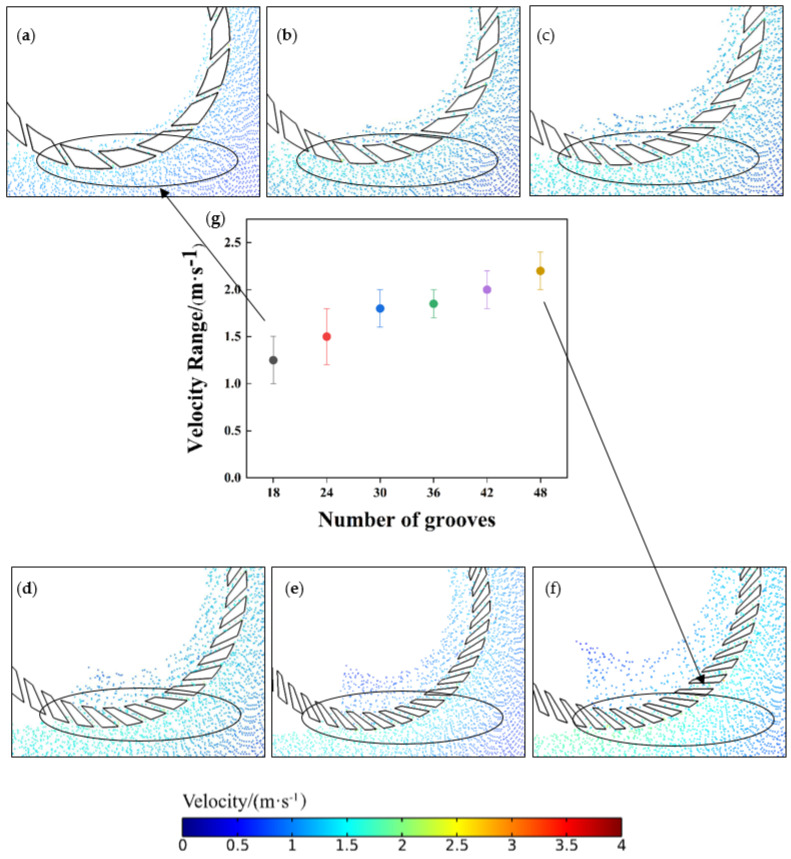
Nephogram of the abrasive speed gradient at the entrance of the ring was maintained with different numbers of grooves: (**a**) 18, (**b**) 24, (**c**) 30, (**d**) 36, (**e**) 42, (**f**) 48. Range of abrasive velocities at the position of the retaining ring abrasive entrance with different groove numbers: (**g**) Range of abrasive velocities at the position of the retaining ring particle entrance with different groove numbers.

As shown above, the number of grooves in the retaining ring affects the abrasive distribution, and the abrasive distribution tends to move closer to the wafer center as the number of grooves increases. The speed of the abrasive entering the retaining ring is an important factor affecting its distribution. Figure 12 traces the abrasive motion at the entrance of the retaining ring at 0.2 s. It shows that the number of grooves affects the speed of the abrasive around the retaining ring. As the number of grooves increases from 18 to 48, the speed of the abrasive increases from 1–1.5 m·s^−1^ for 18 grooves to 1.8–2.2 m·s^−1^ for 48 grooves. Because abrasives have more kinetic energy, the displacement distance of the abrasives is increased. When the number of grooves is increased from 18 to 48, the abrasives are able to move closer to the center of the wafer. This reduces the non-uniformity caused by a high concentration of abrasive at the wafer edge. Therefore, the increase in the number of retaining ring grooves will improve the distribution of abrasives and reduce the unevenness of the wafer surface.

### 4.3. Comparison of the Experiments and Simulations

**Figure 13:**
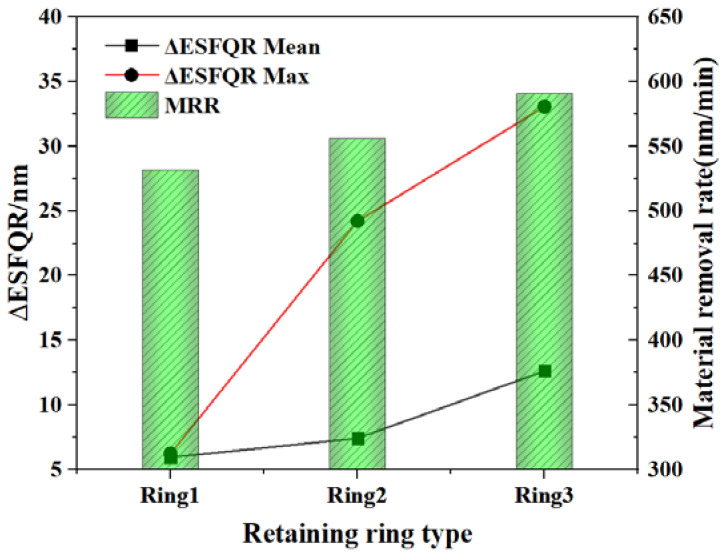
The material removal rate and non-uniformity of 300 mm silicon wafers polished with the Ring1, Ring2, and Ring3.

The simulation results show that the structure of the retaining ring groove will affect the abrasive transfer rate and the abrasive distribution in the wafer region. In order to verify the simulation results, three retaining ring structures were chosen to conduct CMP polishing experiments. The material removal rate and ESFQR improvement are shown in Figure 13.

It can be seen that Ring3 has the highest MRR of 597 nm/min, while Ring2 has an MRR of 557 nm/min, and Ring1 has an MRR of 531 nm/min. Therefore, it is clear that both MRR and ESFQR are improved with the increase of retaining ring groove size, indicating that the flatness of the wafer surface is improved. As analyzed in the above simulations, the abrasive transfer rate of Ring3 is greater than that of Ring2 and Ring1. In addition, Ring3 can also help to distribute the abrasive more evenly in the wafer area, so Ring3 has the highest MRR and flatness. The experiment proves the trend of the influence of the retaining ring structure on the abrasive movement state in the simulation. Meanwhile, the conclusion agrees well with the simulation results of Ring1, Ring2, and Ring3 from the perspective of MRR and uniformity. It can also be obtained that the increase in the number of abrasives leads to an increase in MRR, which explains the highest MRR of Ring3. Furthermore, from Ring1 to Ring3, the abrasives are evenly distributed over the entire wafer area, thus achieving the effect of improving ESFQR.

## 5. Conclusions

In this paper, a fluid-particle multi-physics model is established based on the finite element method and the discrete element method, and the effects of the groove width and the number of grooves of the retaining ring on the abrasive transfer rate and the distribution during the CMP process are studied and validated by experiments, and the following conclusions can be drawn:

- Abrasives can either enter or exit through the grooves at different positions of the retaining ring. Proper adjustment of the size and number of grooves of the retaining ring can result in more abrasives entering than exiting, thus improving the utilization rate of the abrasives. Under the influence of the retaining ring, the abrasive transfer rate varies periodically and becomes stable with time. When the groove size is small, the abrasive transmission through the groove and utilization is poor. Increasing the groove size within a certain range can effectively improve the abrasive transfer rate. The abrasives start to have significant distribution in the wafer region when the groove size reaches 7 mm. When the groove size reaches 13 mm, the abrasive inflow is significantly higher than the outflow, and the transfer rate is at a high level in the later stable stage. However, by continuously increasing the retaining ring groove size, the abrasive transfer rate decreases again. This is due to the fact that at a certain point, the contribution of increasing the groove size to abrasive outflow starts to be greater than that of inflow;
- The results of the retaining ring model based on 7 mm groove size show that the abrasive transfer rate increases to varying degrees at the beginning as the number of grooves in the retaining ring increases and peaks at the number of grooves of 42. In addition, increasing the number of grooves results in the abrasive entering the retaining ring at a higher speed and greater displacement of the abrasive towards the wafer center after passing through the grooves, which helps optimize the abrasive distribution.

The above conclusions show that the design of the retaining ring grooves affects the abrasive motion near the retaining ring. Therefore, improving the design of the retaining ring grooves can improve the abrasive transfer rate and optimize the abrasive distribution in slurry, thus improving the polishing removal rate and reducing the wafer surface non-uniformity. It can also enhance the utilization of slurry and save the polishing cost.

## Figures and Tables

**Table 1:** Parameters used in the simulation model.

Parameter	Value
Retaining ring inner diameter d_2_	301 mm
Retaining ring outer diameter d_1_	348 mm
Retaining ring speed	120 rpm·min^−1^
Pad rotation speed	120 rpm·min^−1^
Abrasive quantity N	30,000
Abrasive diameter d_p_	50 nm
Abrasive density ρ_p_	2300 kg·m^−3^
Slurry density	1150 kg·m^−3^
Polishing slurry viscosity µ	0.02 pa·s
Pad diameter D	850 mm
Polishing pad center position coordinates	(0,0)
Retaining ring center position coordinates	(0,180 mm)

**Table 2:** Simulation scheme for retaining ring groove designs.

Width/mm	Number	Width/mm	Number
3	18	7	18
5	18	7	24
7	18	7	30
9	18	7	36
11	18	7	42
13	18	7	48
15	18		

**Table 3:** Experiment scheme for retaining ring groove designs.

Width/mm	Number	Name
3	18	Ring1
7	18	Ring2
13	18	Ring3

## Data Availability

Not applicable.

## Supplementary Materials

The following supporting information can be downloaded at: https://www.mdpi.com/article/10.3390/ma16010062/s1, Video S1: 7mm × 18 groove abrasives motion state.
